# Dalcetrapib and anacetrapib differently impact HDL structure and function in rabbits and monkeys[S]

**DOI:** 10.1194/jlr.M068940

**Published:** 2017-05-17

**Authors:** Mathieu R. Brodeur, David Rhainds, Daniel Charpentier, Teodora Mihalache-Avram, Mélanie Mecteau, Geneviève Brand, Evelyne Chaput, Anne Perez, Eric J. Niesor, Eric Rhéaume, Cyrille Maugeais, Jean-Claude Tardif

**Affiliations:** Montreal Heart Institute,* Montreal, Quebec, Canada; F. Hoffmann-La Roche Ltd.,† Basel, Switzerland; Faculty of Medicine,§ Université de Montréal, Montreal, Quebec, Canada

**Keywords:** cholesteryl ester transfer protein, high density lipoprotein, efflux

## Abstract

Inhibition of cholesteryl ester transfer protein (CETP) increases HDL cholesterol (HDL-C) levels. However, the circulating CETP level varies and the impact of its inhibition in species with high CETP levels on HDL structure and function remains poorly characterized. This study investigated the effects of dalcetrapib and anacetrapib, the two CETP inhibitors (CETPis) currently being tested in large clinical outcome trials, on HDL particle subclass distribution and cholesterol efflux capacity of serum in rabbits and monkeys. New Zealand White rabbits and vervet monkeys received dalcetrapib and anacetrapib. In rabbits, CETPis increased HDL-C, raised small and large α-migrating HDL, and increased ABCA1-induced cholesterol efflux. In vervet monkeys, although anacetrapib produced similar results, dalcetrapib caused opposite effects because the LDL-C level was increased by 42% and HDL-C decreased by 48% (*P* < 0.01). The levels of α- and preβ-HDL were reduced by 16% (*P* < 0.001) and 69% (*P* < 0.01), resulting in a decrease of the serum cholesterol efflux capacity. CETPis modulate the plasma levels of mature and small HDL in vivo and consequently the cholesterol efflux capacity. The opposite effects of dalcetrapib in different species indicate that its impact on HDL metabolism could vary greatly according to the metabolic environment.

HDL cholesterol (HDL-C) levels are inversely correlated with cardiovascular disease (1). This protective effect could be, at least in part, associated with the ability of HDL to remove cholesterol from peripheral tissues through a process called cholesterol efflux, the first step of reverse cholesterol transport (RCT) (2). In light of these concepts, a variety of therapeutic strategies to modify HDL have been tested in clinical settings.

Cholesteryl ester transfer protein (CETP) is a plasma glycoprotein with a molecular mass of 74 kDa, which is secreted primarily by the liver in primates (3) and in rabbits (4). In vitro CETP activity reduces the HDL-C level by facilitating the transfer of cholesteryl esters (CEs) from HDL to apoB-containing lipoproteins (heterotypic transfer) (3, 5). Accordingly, numerous studies have demonstrated that CETP inhibition increases HDL-C and generates larger mature HDL particles (6–10). Despite the recent disappointing clinical results obtained with the CETP inhibitors (CETPis) (11–13), anacetrapib (NCT01252953) and pharmacogenomics-guided dalcetrapib (14–16) (NCT02525939) are being actively tested in phase 3 clinical outcome trials. In particular, the effects of dalcetrapib on cardiovascular outcome have been shown to vary greatly in different patient populations (14, 16). This strengthens the importance of evaluating the impact of dalcetrapib in species that could show differential responses and be helpful in the comprehension of the effects of dalcetrapib in the various human populations. Thus, a better understanding of the in vivo effects of compounds affecting CETP activity on lipoprotein structure and function is warranted.

HDL remodeling by CETP has been implicated in the production of small lipid-poor preβ-HDL (17, 18). These particles are assumed to be produced by CETP-mediated heterotypic transfer of lipids, but are also produced following transfers among HDL subclasses (homotypic transfer) (19). These small lipid-poor particles are involved in cellular cholesterol efflux (20) via ABCA1 that transports cholesterol and phospholipids to apoA-I (21, 22).

The in vitro inhibition of CETP activity by torcetrapib and anacetrapib is associated with an inhibition of preβ-HDL production, while dalcetrapib preserves this formation (23). However, in vivo studies demonstrated that anacetrapib increased preβ-HDL levels in hamsters, whereas dalcetrapib had no impact on preβ-HDL levels in hamsters (24) and humans (25). Importantly, it was recently demonstrated that administration of anacetrapib to transgenic mice expressing human CETP increased HDL-C and that, in striking contrast, dalcetrapib reduced HDL-C, indicating the importance of studying these CETPis in a model that naturally expresses the CETP. These differences could be generated by the mechanism of action of each CETPi. While anacetrapib generates a nonproductive complex between CETP and HDL, dalcetrapib possesses the unique characteristic of a thioester bond, which is hydrolyzed in its active thiol form. This reactive dalcetrapib-thiol inhibits CETP activity by covalently binding the cysteine 13 of the human protein (9, 26). Interestingly, Morton and Izem (27) recently demonstrated that thimerosal, a thiol-reactive compound that covalently binds cysteines, reduces rabbit CETP activity, but increases hamster, monkey, and human recombinant CETP activity in vitro. They also demonstrated that thimerosal more efficiently inhibits the human and monkey CETP activity transfer in the direction of liposomes containing only triglyceride (TG) compared with liposomes containing CE and TG, while there are no differences between the two types of liposomes when hamster CETP is used. These results suggest that drugs targeting CETP could differentially affect HDL-C and preβ-HDL depending on the mechanism of action and the species involved.

The present study was designed to determine the in vivo impact of dalcetrapib and anacetrapib on HDL subclass distribution in New Zealand White rabbits and vervet monkeys, two species with a high CETP activity level (28) that react in opposite directions to a thiol-reactive compound in vitro. Rabbits and monkeys were exposed to dalcetrapib and anacetrapib and we measured the cholesterol associated to the different lipoprotein classes biochemically and by fast performance LC (FPLC) profiling, the apoA-I distribution among HDL subparticles by nondenaturing gradient gel electrophoresis (NDGGE) and agarose gels, and the ability of apoB-depleted serum to induce cholesterol efflux. We showed that dalcetrapib and anacetrapib substantially increased HDL-C and apoA-I found in large and small HDL subparticles in rabbits, and that these effects were associated with increased cholesterol efflux capacity. In contrast, while similar results were found with anacetrapib in vervet monkeys, dalcetrapib unexpectedly reduced HDL-C levels, apoA-I associated to preβ-HDL, and cholesterol efflux capacities, suggesting that dalcetrapib’s effects on CETP are species dependent.

## MATERIALS AND METHODS

### Materials

The J774 cell line and HepG2 cells were obtained from the ATCC (Manassas, VA). Anacetrapib was bought from Acorn Pharmatech (Redwood City, CA) and dalcetrapib was produced and given by Hoffmann-LaRoche Ltd., Basel, Switzerland.

### Animals and diet

Male New Zealand White rabbits (3.0 kg, aged 12–13 weeks) were first acclimatized for 2 weeks under moderate caloric restriction (∼80% of ad libitum caloric intake) presented as 125 g (or 37.1 g/kg body weight) per day of cholesterol-free diet (32% energy from protein, 13% from fat, and 55% from carbohydrates). Before starting the experimental diet, rabbits were randomized, according to their baseline HDL-C levels, to receive food as above to achieve doses of dalcetrapib of 300 mg/kg body weight (n = 8), or of anacetrapib of 30 mg/kg body weight (n = 7), or food only (n = 7) (supplemental Fig. S1). Food consumption and body weight were recorded throughout the studies to assure adequate drug administration. Blood samples were obtained from an ear vein 1 day before treatment and on day 14 from animals fasted for ≥5 h. Plasma total cholesterol (TC), HDL-C, LDL cholesterol (LDL-C), and TG levels were measured with an automated chemistry analyzer (Dimension RxL Max, Dade Behring, Deerfield, IL). All rabbit experiments were approved by the Animal Care and Use Committee of the Montreal Heart Institute.

Seven female African green monkeys (*Chlorocebus pygerythrus*), also called vervet monkeys, were obtained from a breeding colony and maintained in the primate facility of the South African Medical Research Council (MRC; Cape Town, South Africa) according to the National Code for the Care and Use of Animals for Scientific Purposes and the MRC Ethical Guidelines. The closed indoor environment was maintained at a temperature of 24–26°C, 45% humidity, 15–20 air changes per hour, and a photoperiod of 12 h. Animals were fed a Western-type diet composed of normal human food items with no extra cholesterol added (14% energy from proteins, 47% from lipids, and 39% from carbohydrates; net mean cholesterol intake 23 mg/kg/day), as described in Fincham et al. (29), for over 3 years, except for one individual (over 2 months), prior to the initiation of treatments. Under that diet, TC was increased by 2.6-fold (from 147 to 376 mg/dl) and this increase was principally associated with LDL-C. First, anacetrapib was administered for two consecutive periods of 1 week, starting at 3 mg/kg once daily, and followed by 10 mg/kg once daily. Solutions containing either 12 or 40 mg/ml of anacetrapib in vehicle (soybean oil) were injected (using a syringe) into the center of the food portion. After a washout period of 4 weeks to allow plasmatic parameters to return to baseline values, dalcetrapib was administered at escalating doses for two consecutive periods of 2 weeks, starting at 30 mg/kg three times a day (total dose 90 mg/kg/day) and followed by 60 mg/kg three times a day (total dose 180 mg/kg/day). An exact amount of compound powder, based on the animal’s body weight, was placed into the center of the food portion. Blood samples were collected by femoral venipuncture (after ketamine anesthesia at 5 mg/kg body weight) 3 days before each CETPi treatment and again at the end of each treatment period, 6 h after the intake of the morning portion of food (supplemental Fig. S1). Food consumption and body weight were recorded throughout the studies. HDL-C and LDL-C were determined using standard clinical chemistry methods (Roche Diagnostics). The plasma concentration of compounds was determined using liquid chromatography-tandem mass spectrometry methods that measured the dal-thiol (the active form of dalcetrapib in plasma) (30). Plasma or serum obtained from rabbits or monkeys was stored at −80°C and used within 1 year after freezing.

### CETP mass and activity

Measurement of CETP mass in plasma was performed by ELISA using either mouse monoclonal antibody clone 68/5 or 6/2 as capture antibody for rabbit and monkey, respectively. The detection antibodies were JRC-2 (rabbit) and clone 6/17 (monkey) conjugated to HRP. All of these antibodies were kindly provided by F. Hoffmann-La Roche Ltd.

Rabbit CETP activity was determined by an ex vivo CETP activity assay kit (Roar Biomedical, Inc., New York, NY) (26). Briefly, the fluorescent assays were performed by incubating 95% (v/v) plasma with the commercial reagent up to a total volume of 105 μl at 37°C for 90 min. CETP activity was evaluated in rabbits and monkeys using radiolabeled CE transfer assays, as previously described (23). One microgram of HDL_3_ (1.125 < d < 1.210 g/ml) was labeled with [^3^H]CE and incubated with 65% (v/v) plasma from treated animals in the presence of 1% BSA, 21 mM Tris-HCl (pH 7.4), 0.5% NaCl, and 0.006% EDTA for 4 h (monkey) or 1 h (rabbit) at 37°C. After incubation, the apoB-containing lipoprotein fraction was separated by ultracentrifugation (d = 1.07 g/ml) for 16.5 h at 5°C. Total radioactivity in the apoB-lipoprotein (upper layer) and HDL (lower layer) subfractions were measured by scintillation counting. CETP activity was expressed as the mass (micrograms) of CE transferred to the apoB-lipoprotein fraction per hour. The integrity of HDL_3_ after labeling with CE was verified and confirmed by NDGGE and by size-exclusion chromatography (FPLC) (supplemental Fig. S2).

### Cholesterol distribution in lipoprotein classes by FPLC

Rabbit or vervet plasma was separated by size-exclusion chromatography (FPLC) using a Superose-6 10/300 GL column, as described in (31). The cholesterol content of each fraction was measured with a fluorometric assay.

### NDGGE

The 1D-NDGGE was performed by a modification of the protocol described by Asztalos et al. (32). Briefly, samples were electrophoresed on a 4–30% polyacrylamide gradient gel and run at 150 V for 24 h at 4°C in a buffer containing 90 mM Tris, 80 mM boric acid, and 2.5 mM EDTA (pH 8.3). After electrophoresis, lipoproteins were transferred to a 0.2 μm pore size nitrocellulose membrane (BioRad, Mississauga, Ontario, Canada) at 12 V for 20 h at 4°C in a Tris-glycine buffer without methanol. After transfer, the membrane was blocked and immunodetection was performed with anti-apoA-I-HRP (monoclonal antibody 1/40, provided by F. Hoffmann-La Roche Ltd.) followed by enhanced chemiluminescence detection on Kodak Biomax film.

### Monkey preβ-HDL quantification

Agarose gel electrophoresis was used to determine the preβ-HDL level in monkey plasma. Briefly, after electrophoresis, apoA-I was detected by Western blotting using a HRP-labeled monoclonal antibody against human apoA-I (kindly provided by Dr. Hugues Matile, Hoffmann-La Roche). The apoA-I band corresponding to preβ-HDL migration was identified by comparison with purified human apoA-I.

### Rabbit HDL isolation

Lipoproteins were isolated from rabbit plasma treated or not treated with CETPi. Before isolation, the plasma was adjusted to 0.01% EDTA, 0.02% sodium azide, 10 μM PMSF, and 10 μM butylated hydroxytoluene. HDLs with a density of 1.055–1.21 g/ml were prepared by ultracentrifugation.

### Cell culture

Baby hamster kidney cells expressing a mifepristone-inducible vector with an ABCA1 gene insert (BHK-ABCA1) were a generous gift of Drs. Jack Oram and Chongren Tang (University of Washington, Seattle, WA) (33). BHK-ABCA1 and J774A.1 mouse macrophages were grown in DMEM with phenol red. HepG2 hepatocellular carcinoma cells were growth in Eagle’s minimum essential medium (EMEM). The medium was supplemented with 10% FBS, 100 units/ml penicillin, and 100 μg/ml streptomycin. Cells were cultured in 5% CO_2_ at 37°C and were harvested once a week with trypsin-EDTA. For experiments, cells were trypsinized, seeded, and cultured for 4 days prior to the assays.

### Cellular cholesterol efflux capacity of serum

At confluence, cells were labeled in DMEM (EMEM for HepG2 cells) containing 2 μCi/ml [1,2-^3^H]cholesterol plus 1% FBS for 24 h at 37°C. For J774 cells, 50 μg/ml of human acetylated LDL were also added during the labeling period to generate foam cells. Then, cells were equilibrated with DMEM (EMEM for HepG2 cells) containing 0.2% BSA for 18 h at 37°C with or without 0.3 mM cAMP (J774) or 20 nM mifepristone (BHK-ABCA1) to induce ABCA1 expression. An efflux assay was performed in the absence or presence of 3% rabbit apoB-depleted serum for 4 h, 0.7% vervet monkey apoB-depleted plasma for 6 h, or 50 μg/ml of HDL for BHK-ABCA1, J774, or HepG2 cells, respectively. The apoB-depleted serum was obtained by adding 0.4 vol of polyethylene glycol solution (20% PEG 6000 in 200 mM glycine buffer) to 1 vol of plasma. Samples were vortex-mixed, incubated for 20 min at 4°C, and centrifuged at 10,000 *g* for 30 min at 4°C. At the end of the incubation, the medium was harvested and cells were solubilized. Medium and cells were counted for radioactivity in a β-counter. The percentage of efflux was calculated by subtracting the radioactive counts in the medium in the absence of cholesterol acceptors from the radioactive counts in the presence of acceptor and then dividing by the sum of the radioactive counts in the medium plus the cell fraction.

### Statistical analysis

Statistical analysis was performed independently by the Montreal Health Innovations Coordinating Center statistical analysis group. Data are shown as mean ± SEM unless stated otherwise. Repeated measures ANCOVA models were used for rabbit data, while vervet monkey analyses were done with two-way repeated measures ANOVA.

## RESULTS

### Effects of dalcetrapib and anacetrapib on CETP activity and mass

**Fig. 1.**
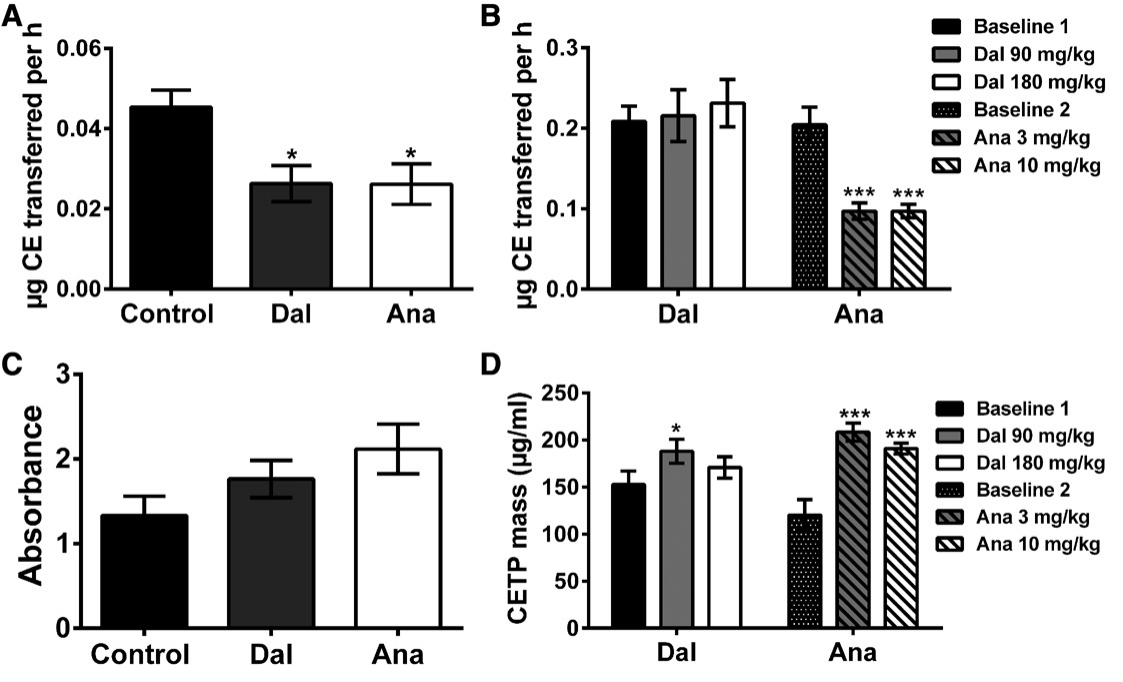
Impact of dalcetrapib (Dal) and anacetrapib (Ana) on rabbit and monkey plasma CETP activity and mass. CETP activity (A, B) and mass (C, D) were evaluated in rabbits (A, C) and monkeys (B, D) treated with dalcetrapib and anacetrapib. At the end of treatment, animal plasma was used for measurement of CETP activity and mass. Results are presented as mean ± SEM of n = 7–8 rabbits and n = 7 monkeys. *P < 0.05, ***P < 0.001 versus baseline values.

To evaluate the level of CETP inhibition, we first measured the activity of CETP in the plasma of control and CETPi-treated rabbits. As shown in **Fig. 1A**, dalcetrapib and anacetrapib both reduced rabbit CETP activity by 42% (*P* < 0.05). To exclude an impact of endogenous lipoproteins, CETP activity was also measured with a commercial kit based on the fluorescent method. Dalcetrapib and anacetrapib caused reductions of CETP activity of 63% and 71%, respectively (supplemental Fig. S3), indicating that the radioactive assay was not affected by the endogenous lipoprotein levels of the samples. In vervet monkeys, anacetrapib significantly reduced CETP activity by 51 and 50% (*P* < 0.001) at 10 and 30 mg/kg/day, respectively, while dalcetrapib did not decrease CETP activity (Fig. 1B). We also determined the impact of CETPi on the levels of circulating CETP. CETP mass was not modified in any group of rabbits (Fig. 1C). In vervet monkeys, CETP mass was significantly increased by dalcetrapib only at 90 mg/kg/day (+23%, *P* < 0.05), while 3 and 10 mg/kg/day of anacetrapib raised it by +74 and +59%, respectively (*P* < 0.001, Fig. 1D).

To confirm the intestinal absorption and systemic exposure to dalcetrapib, pharmacokinetic experiments were conducted in monkeys. Plasma concentrations of the active form of dalcetrapib (dal-thiol) were measured following a single-meal administration. Dal-thiol reached a maximal plasmatic concentration of 1.4 and 5.1 μM, 7 h after single dose administration of 30 and 100 mg/kg, respectively. In rabbits, dal-thiol concentration in plasma was also measured after 14 days of dalcetrapib administration at 300 mg/kg. The concentration of active dal-thiol was 15 μM in animals fasted for ≥5 h. Anacetrapib concentration was also measured in monkeys and the results demonstrated that anacetrapib plasma concentrations reached 1.7 and 2.6 μM after 1 week under administration of 3 and 10 mg/kg of anacetrapib, respectively (data not shown). Thus, it appears that monkeys were exposed to significant concentrations of both CETPis, with differential effects on CETP activity and mass.

### Effects of dalcetrapib and anacetrapib on the lipid profile

**Fig. 2.**
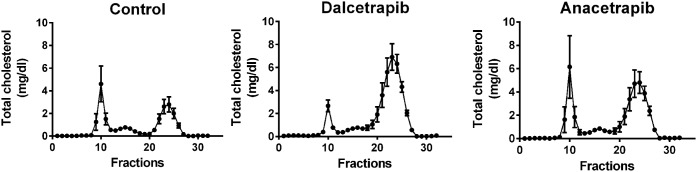
Impact of dalcetrapib and anacetrapib on rabbit FPLC lipid profile. Rabbits were treated with dalcetrapib (300 mg/kg) or anacetrapib (30 mg/kg). At day 14, lipoprotein classes were separated by FPLC and TC concentrations were obtained in each fraction. Results are presented as mean of n = 7–8 rabbits.

**Fig. 3.**
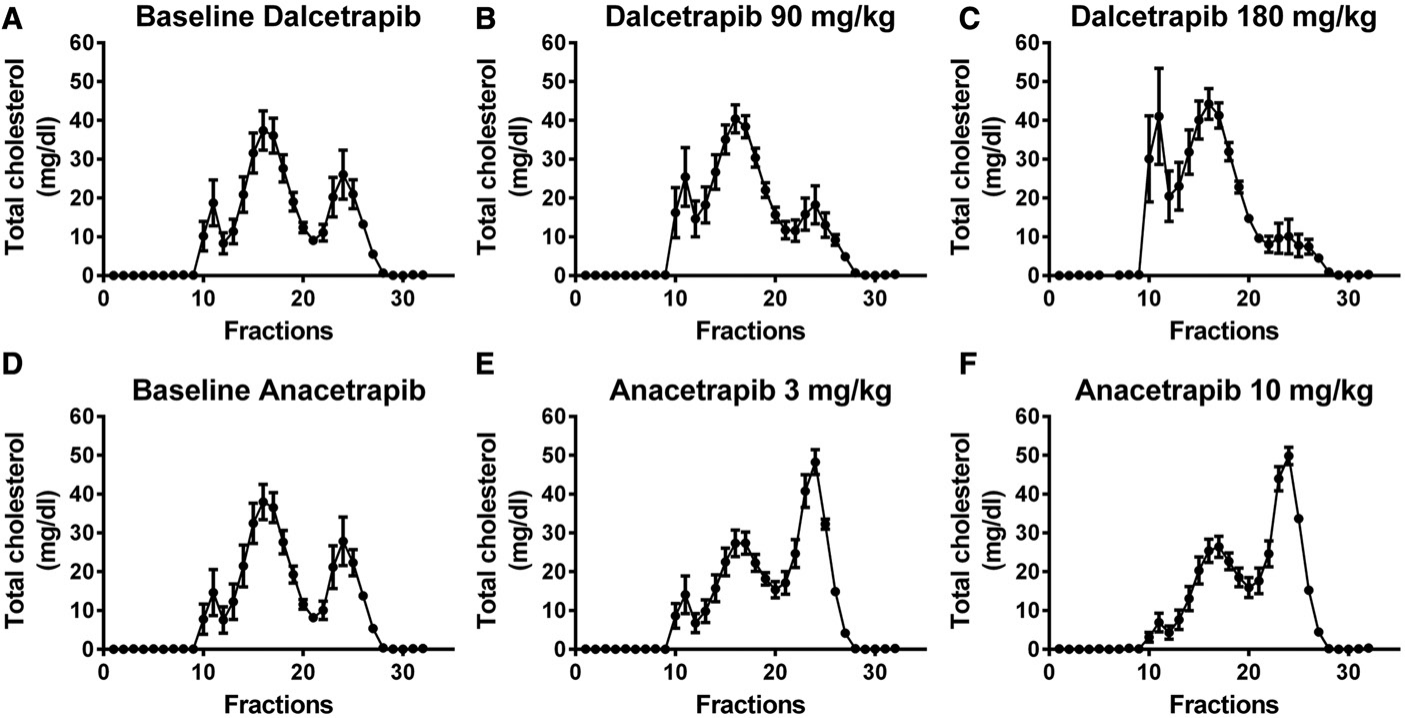
Impact of dalcetrapib and anacetrapib on monkey FPLC lipid profile. Monkeys were treated or not treated with dalcetrapib (90 or 180 mg/kg) (A–C) and anacetrapib (3 or 10 mg/kg) (D–F). Lipoprotein classes were separated by FPLC and TC concentrations were obtained in each fraction. Results are presented as mean of n = 7 monkeys.

Next, we evaluated the impact of CETPis on plasma lipid levels biochemically. The results presented in **Table 1** demonstrate that HDL-C levels were increased by 229% (*P* < 0.01) and 171% (*P* < 0.05) in dalcetrapib- and anacetrapib-treated rabbits, respectively. These higher HDL-C levels were associated with an increase of TC (+86%, *P* < 0.01; +72%, *P* < 0.05), whereas both CETPis had no significant impact on LDL-C. The mean TG level was only increased in the rabbit group receiving anacetrapib (+36%, *P* < 0.01). Accordingly, both CETPis administered to rabbits increased TC in FPLC fractions corresponding to HDL (**Fig. 2**; supplemental Figs. S4, S5), while TC eluting in VLDL or LDL fractions was not modulated. In vervet monkeys, treatment with dalcetrapib resulted in a significant dose-dependent decrease in HDL-C from baseline (−17%, NS and −48%, *P* < 0.01, at 90 and 180 mg/kg/day, respectively) with a significant increase in LDL-C from baseline (+20%, NS; +42%, *P* < 0.01, at 90 and 180 mg/kg/day, respectively; **Table 2**). These decreases in HDL-C and increases in LDL-C, which were also detected by FPLC profiling (**Fig. 3A–C**; supplemental Figs. S6, S7), suggest that dalcetrapib enhances the cholesterol transfer from HDL to apoB-lipoproteins in vervet monkeys. In contrast, anacetrapib induced an increase in HDL-C from baseline (+54% and +59%, *P* < 0.001, at 3 and 10 mg/kg/day, respectively) and a decrease in LDL-C (−16%, *P* < 0.05; −26% *P* < 0.01, at 3 and 10 mg/kg/day) (Table 2). FPLC analysis confirmed these results because cholesterol levels were increased in HDL and reduced in LDL fractions by anacetrapib (Fig. 3D–F; supplemental Figs. S6, S7). Monkey TG levels were unaffected by CETPi treatment.

**TABLE 1.** Impact of CETPi administration on New Zealand White rabbit lipid profile

	Control	Dalcetrapib (300 mg/kg)	Anacetrapib (30 mg/kg)
TC, mmol/l	0.57 ± 0.04	1.06 ± 0.15*^a^*	0.98 ± 0.10*^b^*
HDL-C, mmol/l	0.21 ± 0.04	0.69 ± 0.12*^a^*	0.57 ± 0.11*^b^*
LDL-C, mmol/l	0.14 ± 0.02	0.15 ± 0.02	0.17 ± 0.03
TG, mmol/l	1.08 ± 0.32	0.76 ± 0.10	1.47 ± 0.58*^a^*

Rabbits were fed for 2 weeks with a diet containing dalcetrapib or anacetrapib and lipid profiles were evaluated biochemically. Results are shown as the mean ± SEM of 7–8 animals.

a *P* < 0.01.

b *P* < 0.05.

**TABLE 2.** Impact of CETPi administration on vervet monkey lipid profile

	Dalcetrapib			Anacetrapib		
	Baseline	90 mg/kg	180 mg/kg	Baseline	3 mg/kg	10 mg/kg
HDL-C, mmol/l	2.29 ± 0.34	1.89 ± 0.33	1.19 ± 0.34*^a^*	2.45 ± 0.30	3.78 ± 0.20*^b^*	3.89 ± 0.16*^b^*
LDL-C, mmol/l	5.98 ± 1.00	7.19 ± 0.90	8.52 ± 1.40*^a^*	5.86 ± 0.99	4.93 ± 0.75*^c^*	4.33 ± 0.57*^a^*
TC, mmol/l	8.81 ± 0.90	9.55 ± 0.77	10.35 ± 1.13*^c^*	8.69 ± 0.86	9.58 ± 0.74	9.17 ± 0.60
TG, mmol/l	0.68 ± 0.12	0.55 ± 0.11^*c*^	0.88 ± 0.17	0.66 ± 0.08	0.68 ± 0.07	0.74 ± 0.14

Monkeys were fed a diet containing dalcetrapib or anacetrapib and lipid profiles were evaluated biochemically after 14 and 7 days, respectively. Results are shown as the mean ± SEM of seven animals.

a *P* < 0.01.

b *P* < 0.001.

c *P* < 0.05.

### Effects of dalcetrapib and anacetrapib on apoA-I distribution in HDL subclasses

**Fig. 4.**
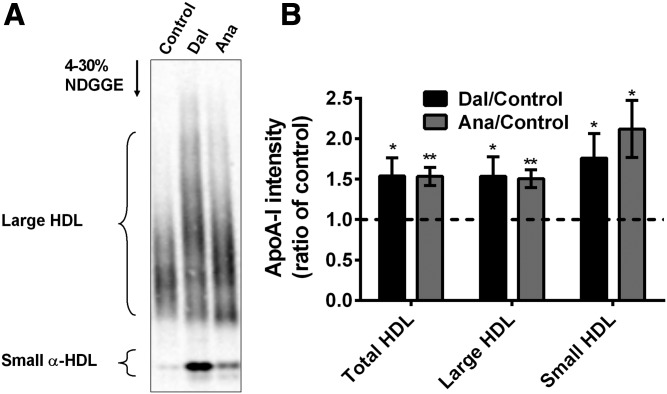
Impact of dalcetrapib (Dal) and anacetrapib (Ana) on rabbit apoA-I distribution in HDL subclasses. Rabbits were fed a control diet (n = 7) or a diet containing dalcetrapib (n = 8) (300 mg/kg) or anacetrapib (n = 7) (30 mg/kg). At day 14, plasma was harvested and used for 4–30% NDGGE. A: Representative Western blot of rabbit plasma apoA-I detection. B: Densitometric analysis of apoA-I Western blots. Ratio calculated from band intensity of each HDL subclass for each drug versus control pair. *P < 0.05, **P < 0.01 versus control.

Initially, we established the distribution of rabbit apoA-I in HDL subclasses by 2D-NDGGE. apoA-I was mainly (93 ± 1%, n = 9) found in large α-migrating HDL of control rabbits. We also found that approximately 6 ± 1% (n = 9) of the rabbit apoA-I was associated to small α-migrating HDL. apoA-I was detected in the preβ position, but it represented only 1 ± 0.3% (n = 9) of total apoA-I (data not shown). Given the low level of apoA-I in preβ position, we analyzed the impact of CETPi on apoA-I distribution in HDL subclasses by 1D-NDGGE. **Figure 4** shows that dalcetrapib and anacetrapib increased total apoA-I associated with HDL (+54%, *P* < 0.05; +54%, *P* < 0.01) in rabbits. This increase was found both in large (+54%, *P* < 0.05; +51%, *P* < 0.01) and small (+76%, *P* < 0.05; +112%, *P* < 0.05) α-migrating HDL (Fig. 4).

**Fig. 5.**
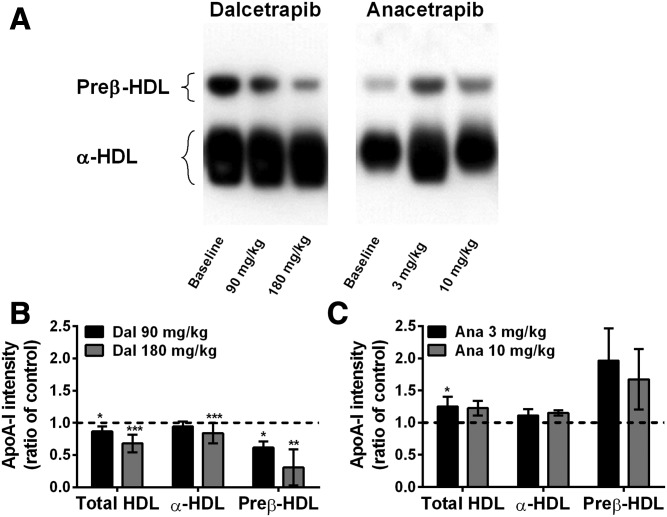
Impact of dalcetrapib (Dal) and anacetrapib (Ana) on vervet monkey apoA-I distribution in HDL subclasses. Monkeys (n = 7) were fed a diet containing dalcetrapib (90 or 180 mg/kg) or anacetrapib (3 or 10 mg/kg). At the end of treatment, plasma was subjected to agarose gel electrophoresis followed by apoA-I Western blot detection. A: Representative Western blot of monkey plasma apoA-I detection. B, C: Densitometric quantification of preβ- and α-HDL levels detected by agarose gel electrophoresis in monkeys receiving dalcetrapib (B) or anacetrapib (C). Ratio calculated from band intensity of each HDL subclass for each drug versus band intensity in the baseline. *P < 0.05, **P < 0.01, ***P < 0.001 versus control.

Agarose gel electrophoresis was used to detect and quantify preβ-HDL of vervet monkeys. Representative images obtained by agarose gel electrophoresis of plasma from dalcetrapib- and anacetrapib-treated monkeys are shown in **Fig. 5A**. At baseline, the apoA-I found in the α- and preβ-migrating positions represented 75 ± 5.6% and 25 ± 5.6% (n = 7) of the total vervet monkey apoA-I, respectively. Densitometric analysis of plasma from dalcetrapib-treated monkeys showed dose-dependent decreases of preβ-HDL (−39%, *P* < 0.05 and −69%, *P* < 0.01, at 90 and 180 mg/kg/day, respectively) and α-HDL (−6%, NS and −16%, *P* < 0.001, at 90 and 180 mg/kg/day, respectively) levels compared with baseline (Fig. 5B). In contrast, although not reaching statistical significance, anacetrapib induced increases of preβ-HDL (+96 and +68%) and α-HDL apoA-I (+11% and +15%) at 3 and 10 mg/kg/day, respectively (Fig. 5C).

### Effects of dalcetrapib and anacetrapib on the HDL remodeling capacity of plasma

**Fig. 6.**
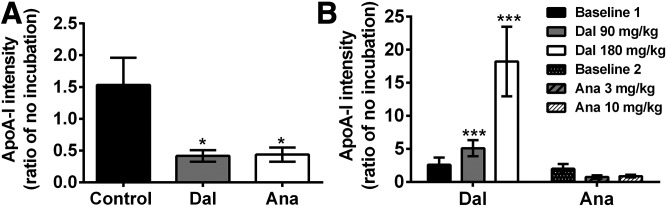
Impact of CETPi on preβ-HDL production in the plasma of rabbits and monkeys. Plasma of rabbits (n = 7–8) (A) or vervet monkeys (n = 7) (B) fed a chow diet or diet containing dalcetrapib (Dal) or anacetrapib (Ana) were incubated at 37°C for 21 h and subjected to 4–30% NDGGE for rabbits or to agarose gel electrophoresis for monkeys, followed by apoA-I detection by Western blot. The ratio was calculated from band intensity of small or preβ-HDL for each drug versus band intensity in the nonincubated samples. *P < 0.05, ***P < 0.001 versus no incubation.

Next, we determined the effects of CETPis on the HDL remodeling capacity of plasma from rabbits and dyslipidemic vervet monkeys. To this end, plasmas were incubated for 21 h at 37°C and 1D-NDGGE or agarose gel electrophoresis was used to detect and quantify small rabbit HDL or monkey preβ-HDL, respectively. **Figure 6A** shows that incubation of control plasma has no effect on the level of small HDL in rabbits. However, the level of small HDL was reduced by 58% (*P* < 0.05) and 56% (*P* < 0.05) in plasma from animals treated with dalcetrapib and anacetrapib, respectively. In monkeys, results showed that the level of preβ-HDL was not modulated in baseline plasma when incubated at 37°C. However, densitometric analysis of incubated plasma from dalcetrapib-treated monkeys showed increased preβ-HDL levels (5.1- and 18.2-fold; *P* < 0.001, at 90 and 180 mg/kg/day, respectively) as compared with corresponding plasma with no incubation (Fig. 6B), demonstrating that dalcetrapib-treated monkey plasma has an increased HDL remodeling capacity. In contrast, preβ-HDL generation upon incubation at 37°C was unaffected by the presence of anacetrapib (Fig. 6B).

### Effects of dalcetrapib and anacetrapib on cholesterol efflux

**Fig. 7.**
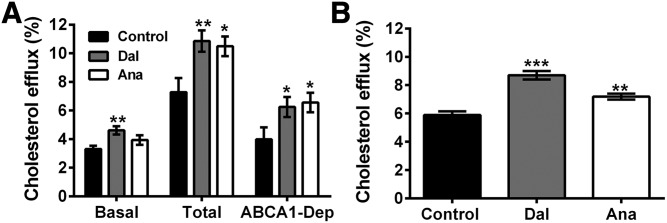
Impact of dalcetrapib (Dal) and anacetrapib (Ana) on cholesterol efflux capacity of rabbit serum and isolated HDL. Rabbits were fed with control diet (n = 7) or diet containing dalcetrapib (n = 8) or anacetrapib (n = 7) and 3% apoB-depleted serum (A) or 50 μg protein/ml HDL (B) was used for cholesterol efflux assays with BHK-ABCA1 cells or HepG2 cells, respectively. apoB-depleted serum was incubated with BHK-ABCA1 cells treated (total efflux) or not treated (basal efflux) with 20 nM mifepristone to induce ABCA1 expression. ABCA1-dependent (ABCA1-Dep) efflux was calculated by subtracting the basal efflux from the total efflux. *P < 0.05, **P < 0.01, ***P < 0.001 versus control.

To assess whether the modulation of apoA-I distribution by CETPis had an impact on cholesterol efflux induced by HDL, we tested the capacity of apoB-depleted serum to accept cholesterol from BHK cells expressing or not expressing ABCA1. **Figure 7A** shows that basal cholesterol efflux induced by apoB-depleted serum from rabbits treated with dalcetrapib or anacetrapib increased by +40% (*P* < 0.01) and +19%, respectively. Moreover, ABCA1-dependent cholesterol efflux from ABCA1-stimulated BHK cells was increased by 57% (*P* < 0.05) and 64% (*P* < 0.05) with these agents (Fig. 7A). Cholesterol efflux was also conducted with HDL isolated from rabbits treated or not treated with CETPis in HepG2 cells and the results show that dalcetrapib and anacetrapib increased cholesterol efflux by 48% (*P* < 0.001) and 22% (*P* < 0.01), respectively (Fig. 7B).

**Fig. 8.**
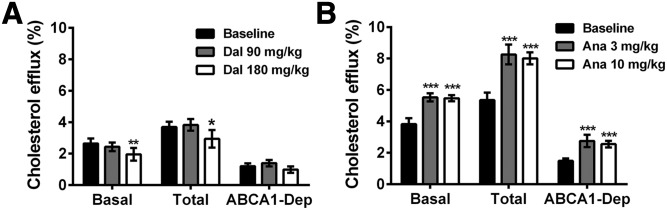
Impact of dalcetrapib (Dal) and anacetrapib (Ana) on cholesterol efflux capacity of monkey serum. Monkeys (n = 7) were fed a diet containing dalcetrapib (A) or anacetrapib (B). apoB-depleted serum (0.7%) was used for cholesterol efflux assays with AcLDL-loaded J774 mouse macrophages treated (total) or not treated (basal) with 0.3 mM cAMP to induce ABCA1 expression. ABCA1-dependent (ABCA1-Dep) efflux was calculated by subtracting the basal efflux from the total efflux. *P < 0.05, **P < 0.01, ***P < 0.001 versus baseline values.

For vervet monkeys, cholesterol efflux capacity was evaluated with AcLDL-loaded J774 macrophages exposed or not exposed to cAMP to induce ABCA1 expression. Basal cholesterol efflux induced by apoB-depleted plasma was not modified by dalcetrapib (90 mg/kg/day) in monkeys, but decreased by 26% (*P* < 0.01) with 180 mg/kg/day (**Fig. 8A**), while anacetrapib-treated monkeys presented a marked increase (+44% and +43%, *P* < 0.001 at 3 and 10 mg/kg/day, respectively, Fig. 8B). These results suggest that the opposite modulation of HDL-C level by dalcetrapib and anacetrapib is also associated with opposite changes in cholesterol efflux. For ABCA1-stimulated cells, although TC efflux induced by dalcetrapib-treated monkey apoB-depleted plasma was decreased by 23% (*P* < 0.05) at 180 mg/kg/day, the ABCA1-dependent efflux was not decreased significantly. In contrast, anacetrapib-treated monkeys presented a marked increase of ABCA1-stimulated TC efflux (+54% and +50%, *P* < 0.001 at 3 and 10 mg/kg/day, respectively), resulting in a significant increase of ABCA1-dependent efflux (+85% and +71%, *P* < 0.001 at 3 and 10 mg/kg/day, respectively, Fig. 8B).

## DISCUSSION

Many studies have demonstrated that HDL-C is a strong predictor of coronary heart disease risk and this inverse association has fostered the development of pharmacological strategies to increase HDL-C levels (34, 35). However, several clinical trials testing drugs targeting HDL have yielded disappointing results (11–13, 36, 37). Nevertheless, large clinical outcomes trials of anacetrapib (NCT01252953) and pharmacogenomics-guided dalcetrapib (NCT02525939) (14–16) are ongoing and contribute to the impetus for the characterization of the effects of these two CETPis on the structure and function of HDL after in vivo administration. Because it was recently demonstrated that thimerosal, a thiol-reactive compound, differently regulated CETP activity according to the species, we studied the impact of CETPis in two animal models that responded in vitro to this compound in opposite directions. Indeed, we used rabbits and nonhuman primates, for which binding of CETP cysteines by thimerosal reduces or stimulates CE transfer, respectively (27). The results from the current study demonstrate that, in vivo, CETPis differently modulate the level of small and mature HDL in these two species and consequently their ability to induce in vitro cholesterol efflux.

Previous studies have demonstrated that HDL particle size has an impact on the catabolism of its apoA-I. Indeed, it was suggested that small dense HDLs are cleared faster than the larger HDLs (38). It was also demonstrated in humans by Brousseau et al. (39) that torcetrapib increases both HDL-C and apoA-I level by reducing its clearance rate. Thus, as expected, gel electrophoresis demonstrated that administration of dalcetrapib and anacetrapib for 2 weeks in rabbits increased the total apoA-I in circulation. A similar effect of anacetrapib was also found in vervet monkeys. Unexpectedly, however, dalcetrapib reduced the level of apoA-I and HDL-C in vervet monkeys. One possible explanation for this reduction could be a decrease in the hepatic or intestinal production of apoA-I induced by dalcetrapib that would be specific to vervet monkeys. Alternatively, this effect might be explained by increased clearance of apoA-I following their enhanced shedding from mature HDL. Previous studies have demonstrated that, in vitro, human preβ-HDL production is stimulated by CETP, because homotypic transfer of cholesterol by CETP among HDL subparticles is enhanced and known to induce the appearance of preβ-HDL (23). Accordingly, we found that incubation of dalcetrapib-treated vervet monkey plasma at 37°C increased the level of preβ-HDL, suggesting CETP activation and HDL remodeling toward the generation of preβ-HDL. In contrast, we found that incubation of CETPi-treated rabbit plasma at 37°C was associated with a reduction of the level of small HDL, suggesting a net maturation of these particles in larger HDL under CETP inhibition. This higher rate of preβ-HDL production in monkeys treated with dalcetrapib could also be linked with the decreased HDL-C level observed in vivo in monkeys. This HDL-C reduction could indeed be explained by increased CETP activity in the presence of dalcetrapib in monkeys, because the decrease in HDL-C was associated with an increase of VLDL- and LDL-C levels. Accordingly, it was recently demonstrated that thimerosal, a compound that, similarly to dalcetrapib, can bind to cysteine residues, activates monkey CETP transfer activity (27). However, the mechanism by which thimerosal acts to increase monkey CETP activity remains to be completely understood. As it is well known that dalcetrapib inhibits CETP activity by interacting with cysteine 13 of the protein (26), one possible explanation is that thiol-reactive (thimerosal or dalcetrapib) compounds interact with the homologous monkey CETP cysteine to increase, instead of reduce, CETP activity, or interact with another cysteine residue. Oppositely, it seems that the action of anacetrapib on CETP from different species is conserved because similar effects were found in rabbits and monkeys. Nevertheless, detailed studies should be undertaken to determine whether the interaction of dalcetrapib with cysteines of CETP differs between the species.

Although the reduction of HDL-C and the enhanced capacity of preβ-HDL generation in monkeys suggest that dalcetrapib increases CETP activity, we did not detect modulation of CETP activity in plasma from dalcetrapib-treated monkeys. It remains possible that the absence of lipoprotein metabolism in the assay does not allow detection of a possible increase of CETP activity. Alternatively, dalcetrapib could be unstable in monkey plasma. However, our results demonstrated that the active form of dalcetrapib (dal-thiol) was detected in monkey plasma, in agreement with previous studies that also found the dal-thiol in the plasma of dalcetrapib-treated monkeys (40, 41). It was recently demonstrated that dalcetrapib reduces the level of HDL-C in wild-type mice (42), a species without any CETP expression. Accordingly, Rios et al. (43) also found that dalcetrapib has CETP-independent effects. Indeed, they demonstrated that dalcetrapib induces the secretion of aldosterone in mouse adipocytes (43) and increases the vascular contraction of rat mesenteric arteries (44). Thus, it remains possible that dalcetrapib could have an off-target effect that could be species dependent.

As demonstrated by FPLC fractionation, anacetrapib (in rabbits and monkeys) and dalcetrapib (in rabbits) increase the cholesterol associated with the HDL fraction, suggesting an improved anti-atherogenic lipid profile. However, evaluation of HDL functionality could provide a better indication of the anti-atherogenic properties of the HDL produced under CETP inhibition in the two species. Thus, we evaluated the impact of these two CETPis on cholesterol efflux induced by apoB-depleted serum to selectively determine the impact of CETPis on HDL without interference by other lipoproteins in the serum. Indeed, apoB-containing lipoproteins could greatly affect cholesterol efflux results because it was previously demonstrated that cholesterol uptake is higher than cholesterol efflux when cells are incubated with LDL, leading to net cholesterol uptake (45). Thus, the presence of apoB-containing lipoproteins in the efflux medium will favor net movement of cholesterol into the cells. Moreover, apoB-containing lipoproteins could confound the cholesterol efflux value because they can accept cholesterol from HDL through the action of CETP. This aspect is particularly important in our assays, given the inhibition of CETP in the treated group only and the possible different levels of LDL particles in the monkeys. Therefore, to selectively evaluate the functionality of HDL, we removed the apoB-containing lipoproteins. The results demonstrated that basal cholesterol efflux mediated by apoB-depleted serum from CETPi-treated animals showing a rise of HDL-C was increased. However, these results give less information about the pathways responsible for that increase. Previously de la Llera-Moya et al. (46) showed a positive correlation between the level of preβ-HDL and ABCA1-dependent cholesterol efflux. As small HDL (rabbits) or preβ-HDL (monkeys) particles were increased by CETPis (with the exception of dalcetrapib in monkeys), we also studied the ABCA1-induced cholesterol efflux. The increase of small HDL particles in plasma was associated with an increase of ABCA1-induced (total) and ABCA1-dependent efflux. In contrast, the decrease of preβ-HDL in monkeys treated with dalcetrapib was accompanied by decreased total efflux that did not result in a significant decrease of ABCA1-dependent efflux, suggesting that the reduction of preβ-HDL induced by dalcetrapib in monkeys was not sufficient to affect ABCA1-dependent efflux. To selectively study the function of HDL particles, isolated HDLs from rabbits treated or not treated with CETPis were used in cholesterol efflux assays with HepG2 cells, a model known to express high levels of SR-BI (47). CETPis increased the capacity of HDLs to induce cholesterol efflux. Given the implication of SR-BI in cholesterol efflux toward mature HDL (48), these results suggest that HDLs produced by dalcetrapib or anacetrapib treatment are more efficient to accept cholesterol. Overall, these results indicate that HDLs produced by CETPi remain functional for the first step of RCT.

As the preβ-HDL particles play an important role in the first step of RCT, the increase of small α-HDL in rabbits or preβ-HDL in monkeys induced by CETPi could be cardioprotective. However, preβ-HDL has also been shown to be increased in patients with cardiovascular diseases (49, 50), suggesting that accumulation of small HDL particles could also be detrimental. The increase of preβ-HDL is dependent on many factors, including a defect in HDL maturation. Indeed, it was demonstrated that deficiency of human LCAT activity is associated with a reduction of HDL-C level and accumulation of discoidal HDL particles in circulation (51). Therefore, the simultaneous presence of low LCAT activity and high preβ-HDL constitutes a strong marker of cardiovascular risk (50). The results of the present study show that accumulation of small HDL is probably not associated with a defect in the maturation of HDL, because we also demonstrated the presence of large HDL particles and increased ABCA1-dependent cholesterol efflux. Thus, anacetrapib (monkeys and rabbits) and dalcetrapib (rabbits) resulted in a new steady-state where all types of HDL particles were increased, allowing the accumulation of a higher level of cholesterol in these particles. In contrast, in monkeys treated with dalcetrapib, preβ-HDL could be cleared from the circulation following enhanced HDL remodeling.

In summary, this study demonstrated that CETPis modulate the in vivo levels of both HDL-C and preβ-HDL and in vitro cholesterol efflux capacity, but these effects differ between species and inhibitors. The opposite effects of dalcetrapib in different species are in sharp contrast to anacetrapib’s responses and indicate that its impact on HDL metabolism could vary greatly according to the metabolic environment.

## Supplementary Material

Supplemental Data
