# Supplementary material for: Dalcetrapib and anacetrapib differently impact HDL structure and function in rabbits and monkeys
Source: J Lipid Res. 2017 May 17;58(7):1282–91. doi: 10.1194/jlr.M068940 (PMC5496027; doi:10.1194/jlr.M068940)
Supplement: Supplemental Data [file supp_58_7_1282__index.html]

Dalcetrapib and anacetrapib differently impact HDL structure and function in rabbits and monkeys — Dalcetrapib and anacetrapib differently impact HDL structure and function in rabbits and monkeys — Supplemental Data 

# Dalcetrapib and anacetrapib differently impact HDL structure and function in rabbits and monkeys

## Supplemental Data

- Supplementary figures (.pdf, 483 KB) - Suplementary figures 1 to 7
