# Supplementary material for: Dalcetrapib and anacetrapib differently impact HDL structure and function in rabbits and monkeys
Source: J Lipid Res. 2017 May 17;58(7):1282–91. doi: 10.1194/jlr.M068940 (PMC5496027; doi:10.1194/jlr.M068940)

Supplementary figures

Supplemental Fig. S1: Schematic representation of experimental design in (A) rabbit and (B) monkey studies. For the rabbit study, animals were acclimatized for 2 weeks under moderate caloric restriction. Then, rabbits were randomized, according to their baseline HDL-C levels, to receive 300 mg/kg/day of dalcetrapib (n = 8), 30 mg/kg/day of anacetrapib (n = 7) or chow diet (n = 7) for the control group. Blood samples were obtained from the marginal ear vein 1 day before treatment start and on day 14 from animals fasted for ≥5 h. For monkeys, animals (n=7) were fed a Western-type diet for over 3 years, except for one individual (over 2 months). First, anacetrapib was incorporated in food for two consecutive periods of 1 week, starting at 3 mg/kg, and followed by 10 mg/kg once daily. After a washout period of 4 weeks, dalcetrapib was administered at escalating doses for two consecutive periods of 2 weeks, starting at 90 mg/kg/day, and followed by 180 mg/kg/day. Blood samples were collected by femoral venipuncture 3 days before each CETPi treatment, and again at the end of each treatment period.

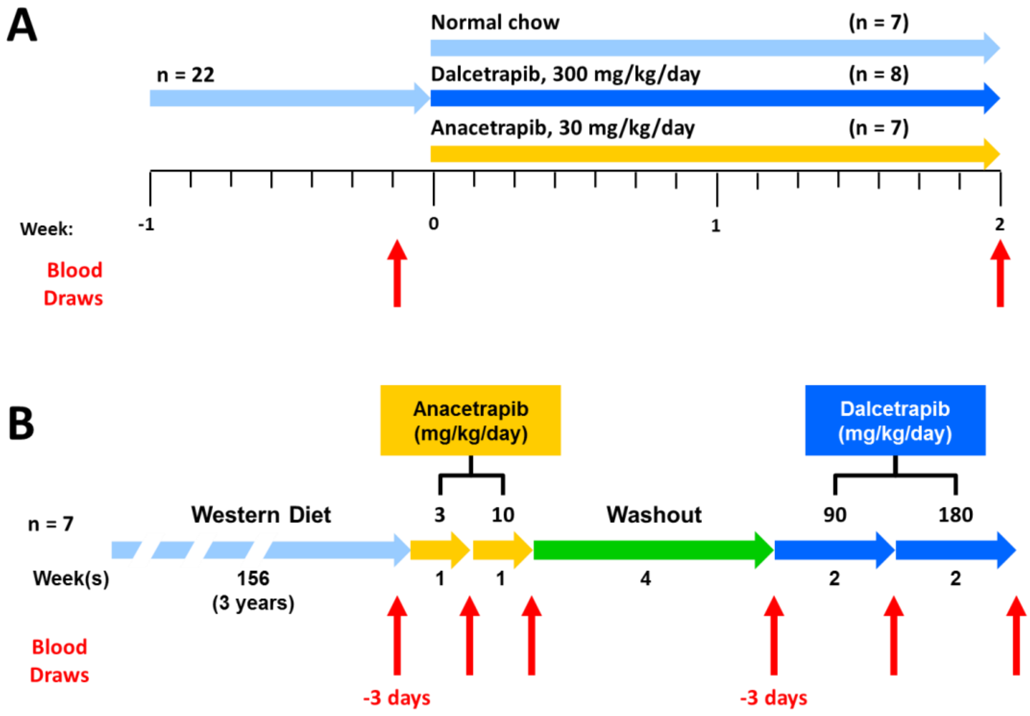

Supplemental Fig. S2: Impact of ^{3}H-CE radiolabeling on the integrity of HDL_3. HDL_3 labeled with radioactive cholesteryl oleate (^{3}H-CE) or with non-radioactive cholesteryl oleate (CE) were (A) electrophoresed on a 4 to 30% polyacrylamide gradient gel stained with coomassie blue or (B) separated by size-exclusion chromatography (FPLC), respectively. The non-radioactive CE labeling was done in the exact same conditions as for ^{3}H-CE labeling.

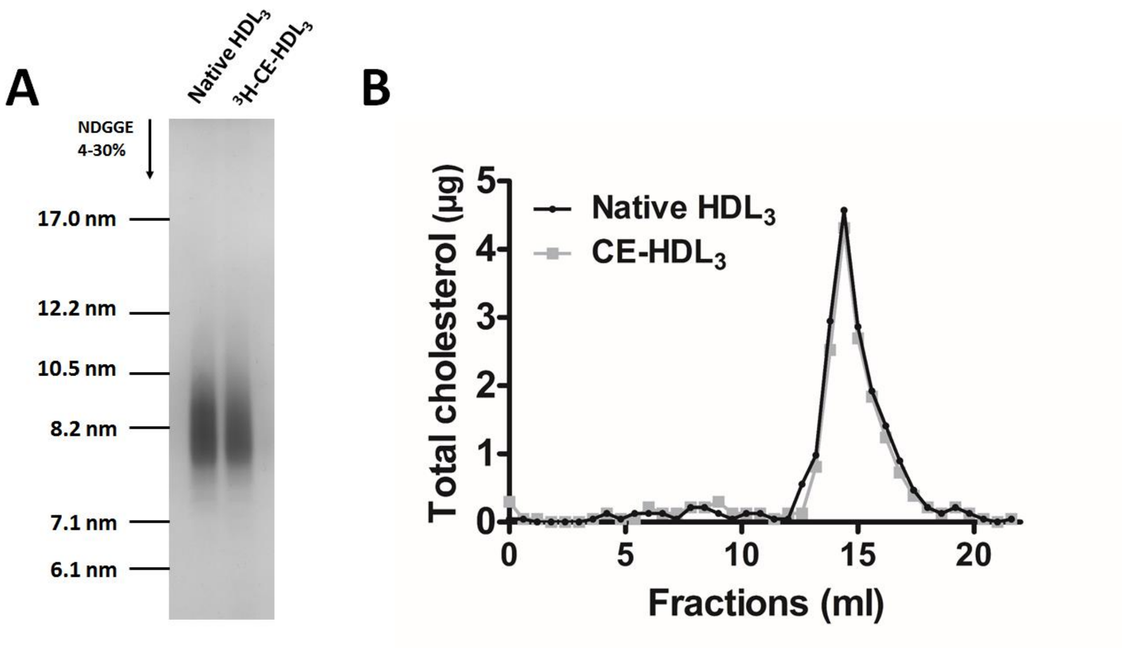

Supplemental Fig. S3: Impact of dalcetrapib and anacetrapib on rabbit plasma CETP activity. Rabbits were treated with dalcetrapib or anacetrapib as described in figure S1. At the end of treatment, CETP activity was measured by the fluorescent method in individual plasma samples. Results are presented as mean ± SEM of 7-8 animals. ** $P < 0.01$, *** $P < 0.001$ versus the control group.

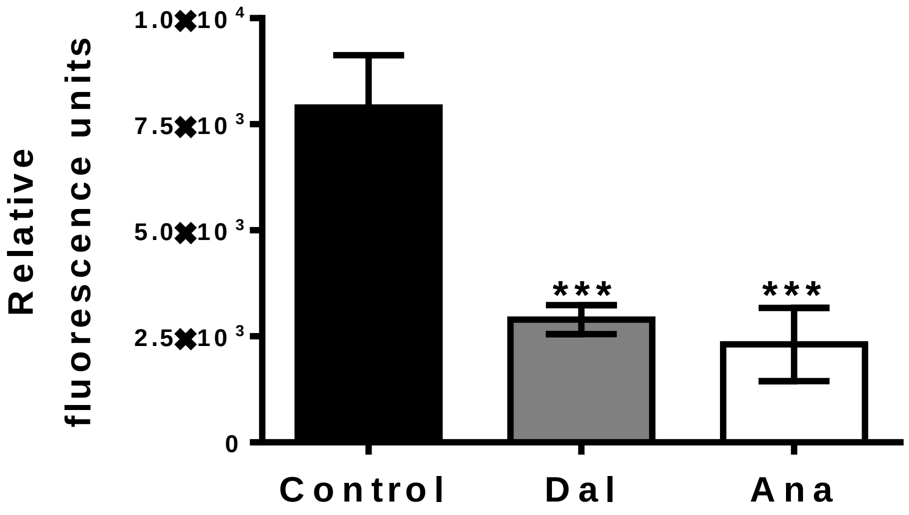

Supplemental Fig. S4: Impact of dalcetrapib and anacetrapib on rabbit FPLC lipid profile. Rabbits were treated with dalcetrapib (300 mg/kg) or anacetrapib (30 mg/kg). On day 14, lipoprotein classes were separated by FPLC and total cholesterol concentrations were obtained in each fraction. Results are presented as mean of n=7-8 rabbits.

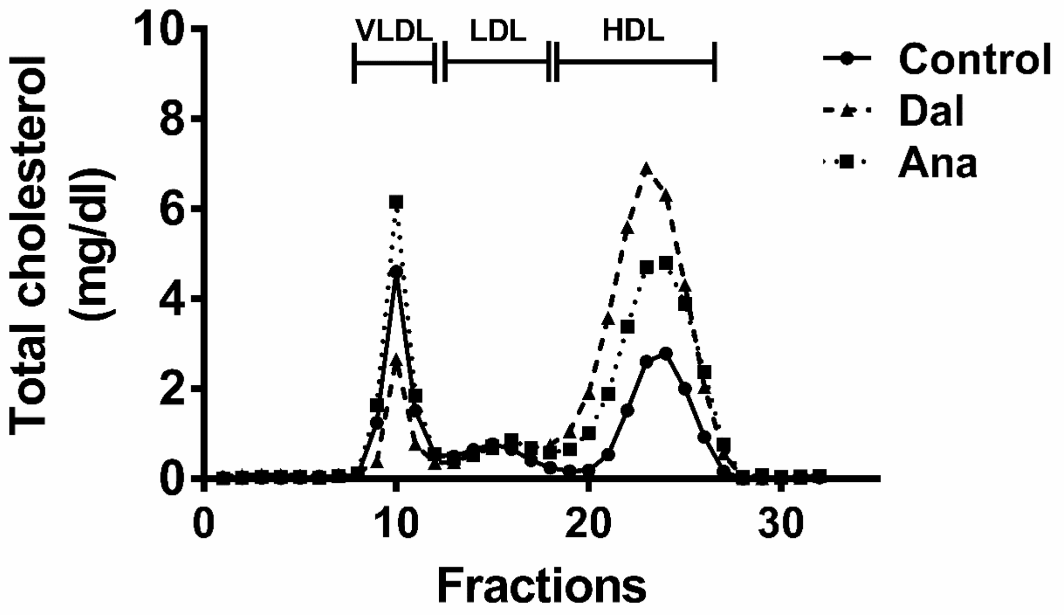

Supplemental Fig. S5: Impact of dalcetrapib and anacetrapib on cholesterol content of lipoprotein classes separated by FPLC from rabbit plasma. Rabbits were treated with dalcetrapib (300 mg/kg) or anacetrapib (30 mg/kg). On day 14, lipoprotein classes were separated by FPLC and total cholesterol concentrations were obtained in each fraction. Cholesterol associated with VLDL, LDL and HDL was evaluated by the sum of cholesterol detected in fraction 8-12, 13-18 and 19-28, respectively. Results are presented as mean of n=7-8 rabbits. * $P < 0.05$, ** $P < 0.01$ versus the control group.

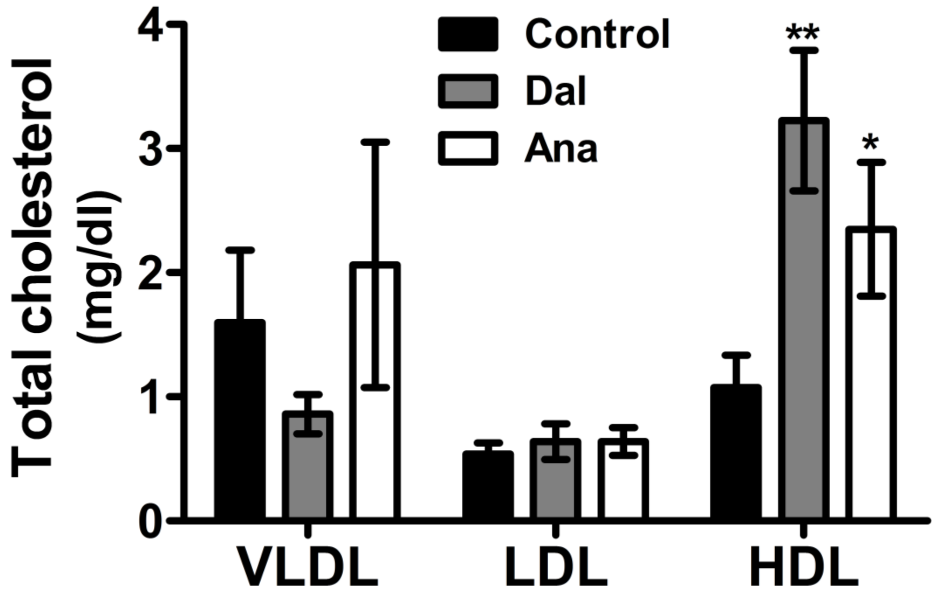

Supplemental Fig. S6: Impact of dalcetrapib and anacetrapib on monkey FPLC lipid profile. Monkeys were treated with (A) dalcetrapib (90 mg/kg or 180 mg/kg) and (B) anacetrapib (3 mg/kg or 10 mg/kg). Lipoprotein classes were separated by FPLC and total cholesterol concentration was obtained in each fraction. Results are presented as mean of n=7 monkeys.

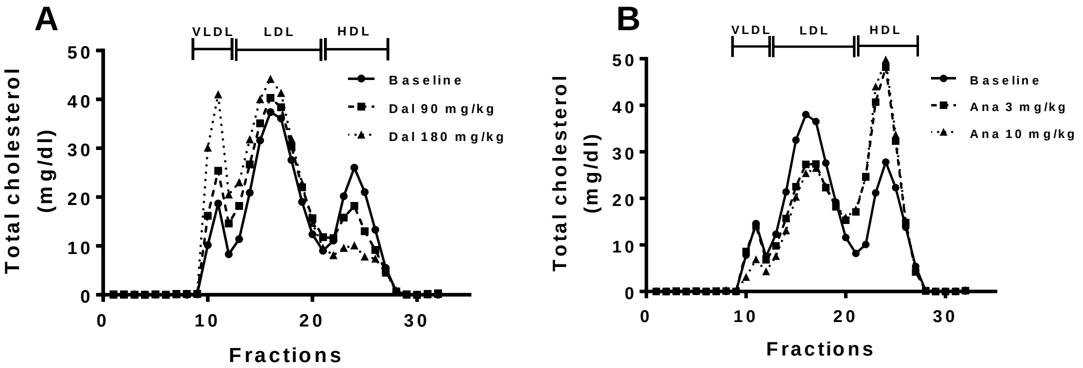

Supplemental Fig. S7: Impact of dalcetrapib and anacetrapib on cholesterol content of lipoprotein classes separated by FPLC from monkey plasma**.** Monkeys were treated with (A) dalcetrapib (90 mg/kg or 180 mg/kg) and (B) anacetrapib (3 mg/kg or 10 mg/kg). Lipoprotein classes were separated by FPLC and total cholesterol concentrations were obtained in each fraction. Cholesterol associated with VLDL, LDL and HDL was evaluated by the sum of cholesterol detected in fraction 9-12, 13-21 and 22-28, respectively. Results are presented as mean of n=7 monkeys. * $P < 0.05$, ** $P < 0.01$, *** $P < 0.001$ versus the control group.

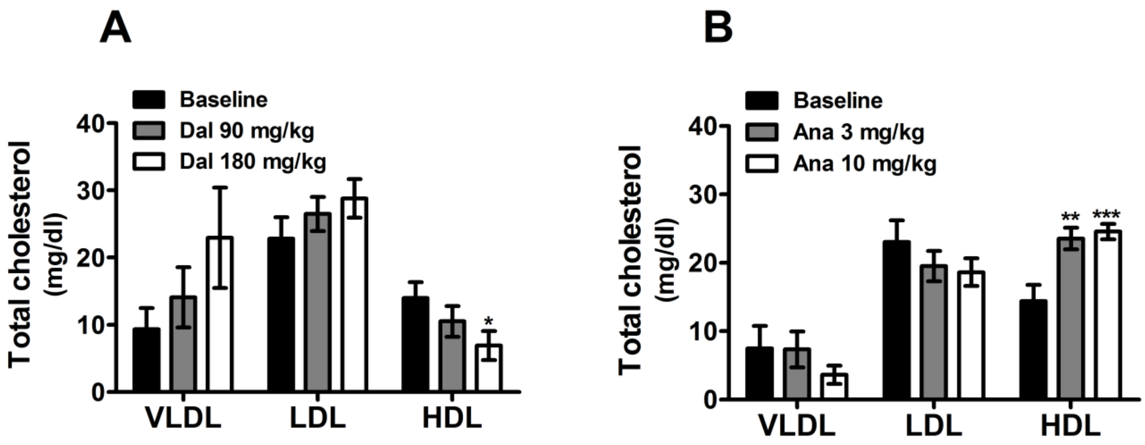

Supplement: Supplemental Data [file 10.1194_M068940_jlr.M068940-1.pdf]
